# Revolutionizing orbital reconstruction: Spectacle -retained prosthesis for post-mucormycosis defect

**DOI:** 10.6026/973206300210456

**Published:** 2025-03-31

**Authors:** Apurva Lambat, Madhav V.N.V., Shreeyash Khadse, Myankita Naru, Lovedeep Singh, Ashish Peter

**Affiliations:** 1 Department of Prosthodontics, YCMM and RDF'S Dental College and Hospital, Ahmednagar, Maharashtra, India; 2 Department of Prosthodontics, Dr. D.Y. Patil Dental College & Hospital, Dr. D.Y. Patil Vidyapeeth, Pimpri, Pune, Maharashtra, India; 3 Department of Dental Surgery, VSPM Dental College and Research Centre, Nagpur, Maharashtra, India; 4 Department of Dental Surgery, Adesh University, Bathinda, Punjab, India; 5 Department of Dental Surgery, Baba Jaswant Singh Dental College and Research Institute, Ludhiana, Punjab, India

**Keywords:** Mucormycosis, orbital prosthesis, enucleation, spectacle retention, customized ocular prosthesis

## Abstract

The incidence of Mucormycosis has been seen an uptrend especially after the COVID-19 epidemic. The number of cases reporting to Prosthodontists has seen an uptick, with increasing demand to rehabilitate the patients with large intraoral and extra oral defects. Mucormycosis is an aggressive fungal infection that can result in severe complications, including involvement of the orbit, often requiring urgent surgical intervention such as enucleation of the affected eye and other structures. This case report details the management of a 57-year-old male patient who had his right eye enucleated due to a Mucormycosis infection two years earlier with a spectacle retained orbital prosthesis.

## Background:

Mucormycosis is a severe and potentially life-threatening fungal infection characterized by its rapid progression and significant complications, one of which may include orbital involvement. In advanced cases of the infection, enucleation of the affected eye often becomes necessary to preserve the patient's life. It is crucial to replace the lost eye as soon as possible after orbital exenteration, as this can significantly enhance the patient's mental and physical recovery and improve social re-integration [1, 2 and 3]. Orbital defects can be devastating to patients not only because of functional impairment of eyesight but also because of the obvious cosmetic deformity. While eyesight cannot be repaired, there have been numerous advances over the past century and past decade to improve aesthetic rehabilitation of orbital injuries [4]. A case report of a 57-year-old male patient who had his right eye removed two years ago due to a Mucormycosis infection is presented in this article. The patient approached the department of Prosthodontics to create an appropriate orbital prosthesis after receiving successful treatment and having enough time to recuperate. A number of criteria, including the patient's preferences, the extent of the defect and anatomical features, have a role in the retention mechanism selection for the orbital prosthesis. In this case, the patient had a deep defect with no undercut areas suitable for conventional retention techniques; therefore other options had to be investigated [1].

## Case report:

A male patient, 57 years old, with history of right eye enucleated was referred to the department of Prosthodontics for rehabilitation. Two years earlier, he had been diagnosed with Mucormycosis. He had completely recovered from the infection and had successfully finished his treatment for Mucormycosis. There were no signs of complications or persistent disease seen during clinical examination. The defect was extensive and devoid of any undercut areas amenable to conventional retention techniques. After discussion with the patient regarding the various treatment options spectacle retained prosthesis was planned as other alternatives such as adhesive retained and implant retained prosthesis were ruled out by the patient [1].

## Methods:

[1] The patient was comfortably seated in the dental chair and explained regarding the impression procedure and the need to record the impression of the face and the contralateral eye.

[2] The defect was blocked with cotton and gauze pack which was coated with petroleum jelly, before making the facial moulage.

[3] A facial moulage using alginate impression was made with help from multiple operators and the impression was strengthened using a plaster outer layer to ensure rigidity to the facial moulage.

[4] The facial moulage was converted to a definitive cast using die stone and the definitive cast was duplicated to retrieve a working cast.

[5] Eosin pencil and graph paper, markings were recorded on the forehead, which were then transferred on to the working casts., in order to help in matching the position and symmetry of the prosthesis with the left eye.

[6] The un-usable undercuts on the working cast were blocked and auto-polymerizing acrylic resin was adapted into the defect in such a way that a small extension was made from the bridge of the nose and to the temple area of the face so that the spectacle frame can be adjusted and adapted later.

[7] The acrylic orbital base was checked with regards to the extension and the fit. Which was then flasked, packed and cured using heat activated acrylic resin to prepare the permanent acrylic orbital base for the final prosthesis.

[8] The permanent acrylic orbital base was retrieved, it was finished and trimmed and was checked for fit and extension.

[9] A readymade stock eye of suitable color match was selected and trimmed to be incorporated into the wax pattern.

[10] The wax pattern was carefully contoured to mimic the shape and form of the contralateral eye, ensuring a natural appearance of the prosthesis. The try in of the wax pattern was done on the patient's face and evaluated, every effort was made to match the esthetics as per the contralateral side.

[11] Subsequently a spectacle frame was selected as per the patient's choice and evaluated with the permanent acrylic base, housing the wax pattern.

[12] Heat temperature vulcanization medical grade silicone (M511 maxillofacial rubber, Technovent Ltd, UK) was mixed according to the manufacturer's instructions, the bonding agent (Cosmesil Platinum Primer G611) was applied to the acrylic base in order to bond the silicone prosthesis to the acrylic base, subsequently the silicone was packed into the flask. Once the prosthesis was cured, it was carefully retrieved and inspected for any irregularities and excess material was cut.

[13] The Acrylic base with the silicone prosthesis was tried on the patient's face. To achieve a close color match, extrinsic staining was done to match with the natural skin tone of the patient.

[14] After achieving the desired results, the spectacle frame was attached to the acrylic base using autopolymerizing resin at the predetermined locations (bridge of the nose and the temple region).

[15] Patient was instructed and trained regarding the use of the prosthesis. Patient was satisfied with the final outcome.

## Discussion:

One of the most traumatic losses of any sense organ is the loss of an eye. It greatly affects a person's sense of self-worth and general well-being. Congenital malformations, trauma, underlying pathology, or tumors can all cause an eye defect. Ocular and orbital prostheses are two categories of eye prostheses. An orbital prosthesis replaces the eye along with the surrounding structures, while an ocular prosthesis replaces the eye artificially without affecting any nearby structures [4, 5-6]. Retention is key to the success of the majority of maxillofacial prostheses, including osseointegrated implants, adhesives, magnets and eyeglass frames. Skin allergies can result from prolonged adhesive use and in order to achieve a high degree of retention, a significant number of supporting substances must be formulated into the adhesive [7, 8]. Magnets can occasionally be used in prostheses [7, 9]. In addition to being an expensive option, long-term use may be hindered by the loss of magnetism or the corrosion of magnets. Osseointegrated implants are the tried-and-true technique for increasing retention, [7, 8, 9, 10-11] nevertheless, this kind of treatment is contraindicated due to a number of reasons, including additional operations, operational expenses, inadequate bone and prior radiation exposure to the region [7, 12]. Retention with eyeglass frames enables for easy fitting of the orbital prosthesis and guarantees accurate reproducible positioning of the restoration, since the slightest error in position will bear visible notice of the prosthesis [7, 13]. Furthermore, spectacle frames act as retentive aid for the acrylic prosthesis to retain the orbital prosthesis. The major advantages of using spectacle retained orbital prosthesis is it being noninvasiveness, no need for surgical intervention, by way of implants, the advantage of utilizing the existing spectacles for retention, no need for any additional adhesives for retention and most importantly, fairly economical to the patient. Considering the patient's desires and the limited resources available, the treatment plan was devised and was executed to achieve optimum results and patient satisfaction in the presented case.

## Conclusion:

Retention in extra oral maxillofacial defects has been mainly achieved through implants, adhesives or magnets. Use of simple retentive aid for the retention of the orbital prosthesis using spectacles in the case presented in this article, helps in minimizing the cost and provides a satisfactory and optimal esthetics, ease in use and patient compliance.
